# The Blue Light‐Responsive Lateral Pathway of the Retinohypothalamic Tract Promotes Endocannabinoid‐Driven Modulation of Orexin Neurons

**DOI:** 10.1111/jnc.70137

**Published:** 2025-06-21

**Authors:** Nicola Forte, Roberta Imperatore, Brenda Marfella, Alessandro Nicois, Roberta Verde, Letizia Palomba, Vincenzo Di Marzo, Luigia Cristino

**Affiliations:** ^1^ Institute of Biomolecular Chemistry National Research Council Pozzuoli Italy; ^2^ Department of Sciences and Technologies University of Sannio Benevento Italy; ^3^ Department of Biology University of Naples Federico II Naples Italy; ^4^ Department of Biomolecular Sciences University of Urbino Carlo Bo Urbino Italy; ^5^ Canada Excellence Research Chair on the Microbiome‐Endocannabinoidome Axis in Metabolic Health Université Laval Québec City Québec Canada; ^6^ Heart and Lung Research Institute of Université Laval Québec City Québec Canada; ^7^ Institute for Nutrition and Functional Foods, Centre NUTRISS Université Laval Québec City Québec Canada

**Keywords:** chronoconnectivity, endocannabinoids, hypocretin, hypothalamus, retinal ganglion cells

## Abstract

Circadian light influences brain functions in mammals. Photic non‐image‐forming stimuli are transduced into electrochemical signals by photosensitive retinal ganglion cells containing melanopsin, a selective blue light‐responsive photopigment. The hypothalamus receives light‐related information via the retinohypothalamic tract (RHT). Here, we demonstrate that, in the mouse, a lateral branch of the RHT (l‐RHT) projects monosynaptically to orexin‐A (OX‐A) neurons in the perifornical hypothalamic area (PFH). Intravitreal injection of the anterograde tracer cholera toxin‐β (CTβ) filled most of the vesicular glutamate transporter (VGluT1)/cannabinoid receptor 1 (CB1R)‐positive retinal‐derived inputs projecting to the OX‐A neurons. Monocular injection of Fluo4‐Dextran, a fluorimetric sensor of calcium mobilization, yielded fast labeling of these inputs 10 min after eye exposure to blue light, concomitantly with the enhancement of hypothalamic 2‐arachidonoylglycerol (2‐AG) levels, and inhibition of OX‐A neuronal firing, an effect prevented by in vivo administration of the CB1R antagonist AM251. Our findings provide anatomical and functional evidence of a selective retino‐hypothalamic network responsive to blue light, whose control should be suitable for therapies to counteract sleep disorders, seasonal affective disorder, or even conditions like narcolepsy or anxiety.

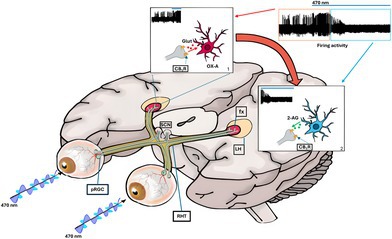

## Graphical abstract

Blue light stimulation of ipRGCs leads to endocannabinoid‐mediated modulation of orexin neuron activity via the retinohypothalamic network. (1) In the first step, blue light‐induced glutamate release from VGluT1/CB1‐positive input depolarizes the OX‐A neuron (red) in the PFH; (2) This depolarization triggers the biosynthesis and release of 2‐AG (green dots), leading to DSE at the retinal VGluT1/CB1R‐positive inputs to the OX‐A neurons, which inhibits further glutamate release and causes hyperpolarization of the OX‐A neurons (blue). ipRGC: photosensitive retinal ganglion cells; OX‐A: orexinergic neuron; RHT: retinohypothalamic tract; fx: perifornical area; LH: lateral hypothalamus; 2‐AG: 2‐arachidonoylglycerol; Glut: Glutamate.

**Abbreviations**
CB1R: cannabinoid receptor
2‐AG: 2‐Arachidonoylglycerol
CTβ: cholera toxin‐β
OX‐A: orexin‐A
PFH: perifornical hypothalamic are
RHT: retinohypothalamic tract
SCN: suprachiasmatic nucleus
VGluT1: vesicular glutamate transporter

## 1 Introduction

Circadian shifts in environmental light frequencies represent the primary “Zeitgeber” by entraining homeostatic brain functions such as eating, sleep/wake cycles, arousal, and hormone production. The mammalian eye contains a non‐image‐forming system constituted by a small group of ipRGCs expressing melanopsin (Provencio et al. 2000, 2002; Hannibal et al. 2002; Berson et al. 2002; Warren et al. 2003), a photopigment maximally sensitive to the blue part of the light spectrum (460–480 nm). The ipRGCs synchronize biological clocks with the external light/dark cycle to regulate the circadian system (Foster et al. 2020). Most ipRGC projections innervate non‐image‐forming hypothalamic areas enrolled in circadian photoentrainment, pupillary light reflexes, and melatonin synthesis (Seabrook et al. 2017; Schmidt et al. 2011). These projections form the retinohypothalamic tract (RHT) (Hannibal 2002) and are mostly glutamatergic, despite a small inhibitory subset regulating the pupillary light reflex (Sonoda et al. 2020). The RHT has been extensively studied in several mammalian species, including mice, using the intravitreal injection of the anterograde‐tracing CTβ (Hannibal and Fahrenkrug 2004; Pickard and Silverman 1981; Johnson et al. 1988; Levine et al. 1991; Murakami et al. 1989; Murakami and Fuller 1990; Mikkelsen 1992; Tessonneaud et al. 1994; Hannibal et al. 1997, 2001). This approach has demonstrated that the RHT innervates the suprachiasmatic nucleus (SCN) bilaterally with approximately equal contralateral and ipsilateral dominance (Hannibal and Fahrenkrug 2004; Abrahamson and Moore 2001). The RHT, in turn, is formed by a medial and lateral retinofugal tract with the lateral part largely overwhelming the medial one into projections to the LH (Leak and Moore 1997), SCN (Welsh et al. 2010), retrochiasmatic and anterior hypothalamic areas (Berson et al. 2002; Hattar et al. 2002; Gooley et al. 2001). The lateral projections (l‐RHT) terminate mainly in the ventral zone of the LH anterior group, including preoptic, lateral, and subparaventricular hypothalamic retinorecipient regions (Hannibal and Fahrenkrug 2004; Hattar et al. 2002; Gooley et al. 2003). Although the medial RHT regulates light entrainment of circadian rhythms to the solar cycle (Hannibal 2002), the function of the l‐RHT is still unknown despite its relevance to the hypothalamic regulation of circadian‐related functions. Here, we focused our study on a putative target of the l‐RHT, a subset of hypothalamic neurons producing Orexin‐A (OX‐A) (Diaz et al. 2023), also called hypocretin‐1. OX‐A is a 33‐amino‐acid peptide that regulates circadian functions such as arousal, feeding, and the sleep–wake cycle. In nocturnal animals like rodents, a melanopsin‐induced activation of OX‐A neurons has been described in association with sleep‐to‐wake transition (Tsunematsu 2021; Deboer et al. 2004; Zhang et al. 2004; Azeez et al. 2018; Ventzke et al. 2019). These data are in line with the circadian changes of cerebrospinal and hypothalamic OX‐A levels, which are increased in the awake phase (i.e., ZT13‐24) and decreased in the resting phase between ZT8 and ZT12, immediately before the onset of the awake phase (Yoshida et al. 2001; Fujiki et al. 2001; Desarnaud et al. 2004).

A circadian daytime‐induced change of the endocannabinoid 2‐AG levels has been described in the rodent hypothalamus (Valenti et al. 2004). Notably, changes in OX‐A levels were also associated with fast non‐genomic 2‐AG‐driven control of orexinergic neurons at CB1R receptors (Cristino et al. 2013). In turn, OX‐A signaling regulates 2‐AG synthesis via OX‐1R receptors, promoting a close relationship between the circadian changes of OX‐A and 2‐AG levels in regulating sleep timing and caloric intake (Cristino et al. 2013; Hanlon et al. 2015, 2016; Cedernaes et al. 2016; Forte et al. 2021). For instance, (i) intra‐LH administration of 2‐AG increases REM sleep, (ii) the CB1R agonist (CP55940) attenuates light‐induced clock‐phase advance (Sanford et al. 2008), and (iii) the cannabinoid Δ9‐THC, the primary psychoactive agent of marijuana and hashish, tends to generate a distorted sense of time by signaling at hypothalamic CB1R (Sewell et al. 2013; Tinklenberg et al. 1976). However, anatomical evidence of an ipRGCs retinofugal projections to OX‐A neurons, as well as the functional evidence of a blue light‐mediated change of OX‐A neuronal activity and 2‐AG levels in the hypothalamic nuclei, are still missing.

## 2 Material and Methods

### 2.1 Animals and Drugs

The study was conducted according to the ARRIVE Guidelines to improve bioscience research reporting using laboratory animals. All efforts were made to minimize animal suffering and to reduce the number of animals used. The experiments were performed by the European Union animal welfare guidelines [European Communities Council Directive of September 22, 2010 (2010/63/EU)] and the Italian Decree no. 26/2014, authorization no. 589/2018‐PR. Eighty‐seven C57Bl/6J male mice (Charles River Laboratories Int.; Wilmington, Massachusetts, USA; 2–3 months old, ~25 g body weight) were housed in standard cages receiving chow and tap water ad libitum under controlled illumination (12 h light/dark cycle; light on at 6:00 am, ZT0) and environmental conditions (ambient temperature 20°C–22°C, humidity ~50%). As orexin levels exhibit robust diurnal fluctuations, gradually increasing between ZT13‐24 (dark period) and decreasing during ZT0‐12 (light period), all the experiments were performed between ZT6 and ZT12, when orexin neurons are less active (Yoshida et al. 2001; Fujiki et al. 2001; Desarnaud et al. 2004). Before the blue light exposure, mice were habituated to total darkness for 1 h starting from ZT5 to maximize this specific wavelength's effect on the ipRGCs stimulation.

### 2.2 Intravitreal Injection of Tracer Cholera Toxin Subunit B or Fluo‐4‐Dextran, and Eye Stimulation

An Intraocular injection was performed into the vitreous of the left eye of adult C57BL/6J mice anaesthesized with a mixture of ketamine (0.08 mg/g body weight; Sigma, cat.no. K2753) and xylazine (0.01 mg/g body weight; Sigma; cat. no. X1251), and topical treatment with proparacaine hydrochloride 0.5% (Akorn, Buffalo Grove, IL, USA) maintained under 12 h:12 h dark/light cycle. Intravitreal injection of anterograde tracer cholera toxin‐β (CTβ, List Biological cat. no., 104, RRID:AB_2313636, Campbell, CA, USA), or Fluo4‐Dextrane (Fluo‐4, Life Technology, cat. no. F14201) was performed in two different groups of mice (no. 6 mice/group) maintained under 12 h:12 h dark/light cycle as described by Prichard et al. (2002). Briefly, a small hole was made in the temporal sclera margin of the eye with a sterile 26‐gauge needle, and 2.0 μL of CTβ dissolved in saline with 0.1% Evans Blue (Sigma) or 2.0 μL of Fluo4‐Dextrane were injected into the vitreous by holding the needle in place for 1 min after administration to prevent tracer's reflux. 7 days after Flou4‐Dex injection, the mice were kept in total darkness for 1 h before being stimulated with a blue light pulse (LP, 800 Lux) (Huang et al. 2023; Bilu et al. 2019) at constant irradiance for 10 or 20 min and then rapidly euthanised and perfused transcardially with 4% PFA.

### 2.3 Immunohistochemistry

Mice were deeply anesthetized with a mixture of ketamine and medetomidine (100 mg/kg + 1 mg/kg i.p.) and perfused transcardially with 4% (wt/vol) paraformaldehyde (SERVA, cat no. 3162802)/0.1 M phosphate buffer (PB), ph 7.4. The brains were extracted and cryo‐preserved by immersion in 30% (wt/vol) sucrose/0.1 M PB until sinking and then cut with a Leica CM3050S cryostat in 10 μm‐thick serial coronal sections collected in alternate series to be processed for immunofluorescence. Sections were incubated for 1 h at room temperature in PB containing 0.3% Triton X‐100 (Sigma, cat no. X100) and 5% normal donkey serum (blocking buffer, Abcam, cat. no. ab7475), and then incubated overnight at 4°C with the following mixture of primary antibodies diluted in donkey serum: mouse anti‐CTβ (Abcam, cat. no. ab62429; 1:500), goat anti‐OX‐A (Santa Cruz, cat. no. SC8070; 1:200), guinea pig anti‐VGluT1 (Synaptic System, cat. no. 135304, 1:500), rabbit anti‐CB1R antibody (anti‐C terminus 461‐472, Abcam, cat. no. ab23703; 1:300), rabbit anti‐c‐Fos (Abcam, cat. no. ab190289; 1:200). Immunofluorescence was revealed by specific Alexa‐488, or − 546, or − 350 secondary donkey anti‐IgGs (Invitrogen, ThermoFisher Scientific, France, cat. no. A10035, A21081, A17655, A21207) incubated 2 h at room temperature at 1:250–1:100 dilution range. Sections were coverslipped with Aquatex mounting medium (Merck, Darmstadt, Germany). Controls of the specificity of immunolabeling in multiple fluorescence experiments were performed by omission of primary and secondary antibodies or by preabsorption of primary antibodies with the respective blocking peptides. The immunostained sections were observed with a confocal microscopy Nikon Eclipse Ti2 (Nikon, Florence, Italy) equipped with an x‐y‐z motorized stage, a digital camera DS‐Qi2 (Nikon, Florence, Italy), and the acquisition and Image analysis software NIS‐Elements C (Nikon, Florence, Italy). Digital images were acquired using the ×20 − ×40 objectives and serial Z‐stacks were collected throughout the area of interest (*n* ≤ 20 planes with an increment of 0.5 μm). Images were deconvolved using the imaging deconvolution software by application of n=10 iterations. Serial Z‐plane images were collapsed into a single maximum projection image. Micrographs were saved in TIFF format and adjusted for light and contrast before being assembled on plates using Adobe Photoshop 6.01 (Adobe Systems, San Jose, CA).

### 2.4 Immunohistochemical Analysis

Assessment of immunoreactivity was performed throughout the anterior–posterior extension of the hypothalamic nucleus, in a series of 10 μm coronal sections spaced 30 μm apart (1:3 as the frequency of section sampling to cell count, starting from Bregma −1.34 mm up to Bregma −2.18 mm), through 0.85 mm^3^ rostrocaudal extension from the 3^rd^ ventricle. For the quantification of single or multiple immunoreactivity and cell counting, an unbiased physical dissector‐based protocol was applied by an operator blind to the experimental groups, according to Forte et al. (2021).

### 2.5 Lipid Extraction and 2‐AG Measurement

PFH of the hypothalami from the brains of mice under the same experimental conditions were accurately dissected and pooled (*n* = 12/group) to be analyzed using liquid chromatography–atmospheric pressure chemical ionization–mass spectrometry. The 2‐AG was extracted from the tissues and then purified and quantified as previously described. First, tissues were pooled and homogenized in 5 vol chloroform/methanol/Tris–HCl 50 mM ph 7.5 (2:1:1 by volume) containing 50 pmol of d5‐2‐arachidonoylglycerol (d5‐2‐AG) as an internal deuterate standard. Homogenates were centrifuged at 13 000 *g* for 16 min (4°C), and the aqueous phase plus debris were collected and extracted four times with 1 vol of chloroform. The lipid‐containing organic phases were dried and pre‐purified by open‐bed chromatography on silica columns eluted with increasing concentrations of methanol in chloroform. Fractions for 2‐AG measurement were obtained by eluting the columns with 9:1 (by volume) chloroform/methanol and then analyzed by liquid chromatography/atmospheric pressure chemical ionization–mass spectrometry (LC‐APCI‐MS). LC‐APCI16 MS analyses were conducted in the selected ion monitoring mode, using *m*/*z* values of 384.35 and 379.35 (molecular ions +1 for deuterated and undeuterated 2‐AG). Values are expressed as pmol per mg of wet tissue extracted.

### 2.6 Electrophysiology

Male C57BL/6J mice, aged 2–3 months, were used throughout all experiments. Mice were anesthetized under urethane (1.2 g/kg, i.p.), oxygen was administered to the animal through a mask, and body temperature was held at 37°C through a thermostatic blanket. During experiments, *lacrigel* was used to prevent corneal dehydration. A bilateral craniotomy of approximately 1 mm was made on the LH. The following stereotaxic coordinates were used to target the LH: anterior/posterior −1 mm from Bregma, lateral ±1 mm, and dorsal/ventral between −4.3 and − 5.3 mm. Single‐unit recordings from unidentified neurons in the LH were acquired using a microelectrode filled with K‐acetate 0.5 M, 6–10 μm at the tip. The recordings were performed using a model 410 amplifier (Brownlee Precision Co) and power 1401 mkII (CED), bandpass (200–3000 Hz). The recording sessions were conducted in the dark. The eye contralateral to the recording site was stimulated using a blue light emitted from LEDD1B (Thor Labs) coupled with a lens (Toptica Photonics, Fiberout_011555). The light power, measured with a power meter 3 cm away from the light source, was approximately 4 mW/mm^2^. At the end of the recording session, animals were perfused with 4% PFA to confirm the correct position of the recording site. All data were analyzed using Spike2 (CED).

Spontaneous neuronal activity in the dark was recorded for 5 min in the PFH, followed by a 10 min ofblue light pulse. Subsequently, additional 5 min of recording in the dark were performed. In a subset of experiments, the CB1R inverse agonist AM251 (Tocris cat no. 1117, 1 mg/kg) was administered intraperitoneally (i.p.) 1 h before the start of the recording session.

### 2.7 Statistical Analysis

Data were analyzed using GraphPad Prism (RRID:SCR_002798). No sample size was calculated a priori, but the sample size was based on previous studies of a similar nature (Forte et al. 2021). No exclusion criteria were pre‐determined, and no blinding was performed. Outliers were detected using the Rout method with Q = 1%. Data were reported as bar or box plots. The box plot elements were center line, median (Q2); square symbol, mean; box limits, 25th (Q1), and 75th (Q3) percentiles; the outermost data points determined whisker length. Normal distribution was assessed using the Shapiro–Wilk normality test. The electrophysiological experiments were analyzed using a one‐sample *T*‐Test and a two‐way ANOVA test. For the neuroanatomical and immunohistochemical experiments, One‐way ANOVA/Bonferroni tests or Kruskal–Wallis/Dunn's tests were used to analyze more than two experimental groups with normally or non‐normally distributed data, respectively. Data are expressed as mean ± SEM.

## 3 Results

### 3.1 Retinal VGluT1/CB1R‐Positive Projections Innervate the Hypothalamic PFH

**FIGURE 1:**
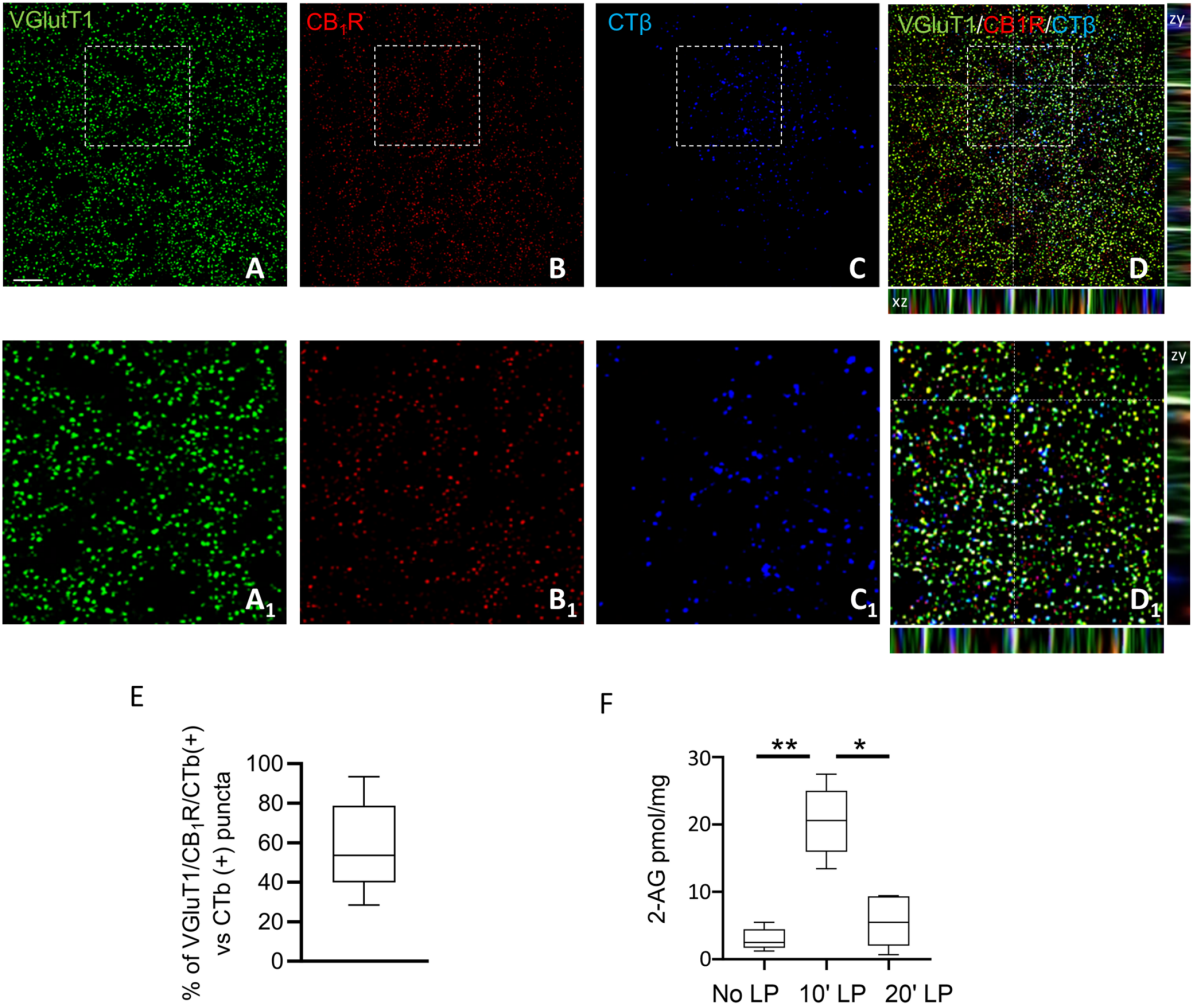
Intravitreal CTβ loading of VGluT1/CB1‐positive inputs to the lateral hypothalamus and blue light‐mediated regulation of 2‐AG levels. (A–C) Confocal microscopy showing retinal fibers labeled with CTβ (blue), expressing vesicular glutamate transporter VGluT1 (green) and CB_1_R (red). Scale bar: 20 μm. (D) High‐power fluorescent micrographs of orthogonal stacks; dotted lines and crosshairs are used to show 3D coordinates and define the area of interest. (A_1_–D_1_) Magnification of the details highlighted in the squares reported in A–D. (E) Bar graph showing the percentage of VGLUT1/CB1R/CTβ − immunoreactive puncta despite to the total number (100%) of CTβ − labeled puncta, *n* = 30 sections/mice; *n* = 3 mice. (F) LC–MS quantification of 2‐AG levels (pmol/mg) in the PFH of mice under different conditions: No LP, 10 min blue light pulse (10′LP), or 20 min blue light pulse (20′LP) exposure. *n* = 10 mice per group. Kruskal–Wallis Test and Dunn's post hoc test, DF = 2, Kruskal–Wallis statistic = 9.893, 30.7, *p = 0.042, **p = 0.0053. Mean ± SEM.

Previous studies have identified RHT projections to the LH in the rat brain by using the anterograde‐tracer CTβ (Hannibal and Fahrenkrug 2004; Levine et al. 1991; Hattar et al. 2002; Gooley et al. 2003). Other studies demonstrated the high expression of CB1R at the pre‐synaptic level in the LH (Cristino et al. 2013; Jo et al. 2005). However, whether CB1Rs are expressed at retinal‐derived hypothalamic inputs is still unknown. To trace the retinal projection to PFH in the LH we used intravitreal injection of CTβ, which does not travel trans‐synaptically (Coolen et al. 1999; Mehlman et al. 2021). CTβ was chosen for its superior uptake, sensitivity, rapid transport, and frequent use in the analysis of the retinocollicular tract (Angelucci et al. 1996). Analyzing the VGluT1 and CB1R immunofluorescence on diencephalic sections from CTβ‐injected brains, we observed a vast majority of retinohypothalamic CTβ‐labeled inputs in the PFH (Figure 1A–D,A_1_–D_1_), most of which expressed VGluT1/CB1R immunoreactivity (Figure 1E).

### 3.2 Eye Blue Light Stimulation Regulates Hypothalamic Endocannabinoid 2‐AG Levels and c‐Fos Expression in OX‐A Neurons

A circadian daytime‐induced change in endocannabinoid levels has been described in rat brain and human plasma (Valenti et al. 2004; Hanlon et al. 2015). In this study, by exploiting mass spectrometry, we report a blue light‐dependent change of endocannabinoid 2‐AG levels in the PFH. Specifically, we observed an almost 10‐fold increase of 2‐AG levels (20.50 ± 2.309 pmol/mg) after 10 min of exposure to blue light, compared to the control group (ins levels; Figure 1F). On the other hand, 20 min of blue light exposure induced a significant reduction of 2‐AG levels in comparison to the levels observed after 10 min of blue light exposure (5.6 ± 3.7 pmol/mg) (Figure 1F).

**FIGURE 2:**
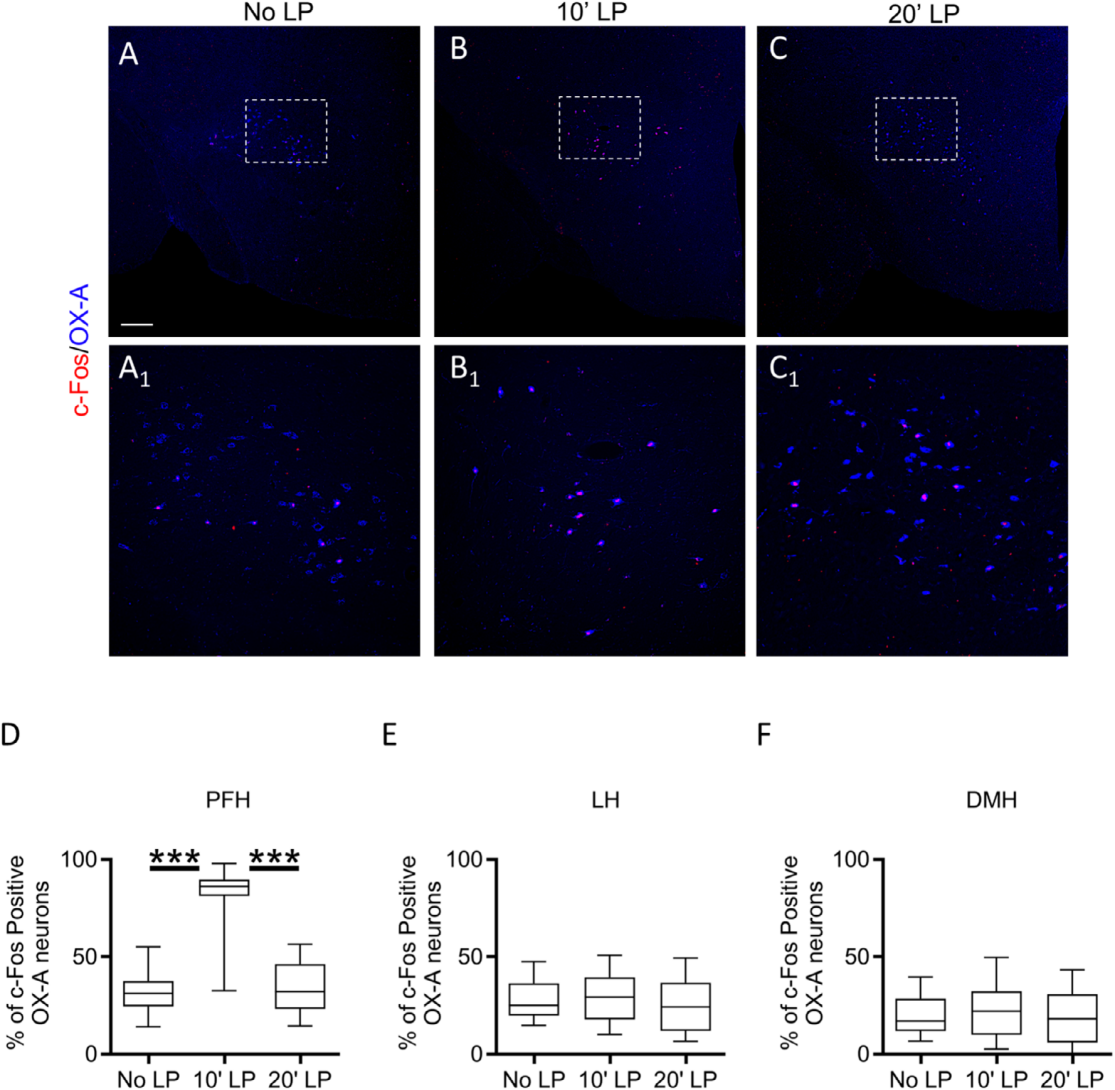
Blue light exposure increases c‐Fos expression in OX−A neurons in the PFH. (A–C) Confocal microscopy showing colocalization of c‐Fos (green) and OX‐A (red) immunoreactivity in the PFH of mice under No LP, 10′LP or 20′LP blue light pulse exposure. Scale bar: 300 μm. (A_1_–C_1_) Magnification of the details highlighted in the squares depicted in A–C. (D–F) Percentage of c‐Fos/OX‐A‐immunoreactive neurons in the PFH, LH, or DMH of mice under No LP, 10′LP, or 20′LP blue light pulse exposure. *n* = 30 sections/mouse; *n* = 6 mice/group. Kruskal–Wallis Test and Dunn's post hoc test, ****p < 0.0001, DF = 2, Kruskal–Wallis statistic = 48.56. Mean ± SEM.

Subsequently, we observed a significant increase in c‐Fos immunoreactivity in OX‐A neurons within the PFH following 10 min of blue light exposure (82.63% ± 2.7% mean ± SEM), compared to the no‐light pulse (No‐LP) condition (32.09% ± 1.9% mean ± SEM; Figure 2A,B,A1–B1,D, neurons). In contrast, no significant difference was detected when the stimulation was extended to 20 min (34.14% ± 2.4% mean ± SEM; Figure 2C–C1,2D). Furthermore, no changes in c‐Fos expression were observed in the LH or DMH after either 10 or 20 min of light stimulation (Figure 2E,F). No variation in the number of OX‐A immunoreactive neurons was detected in No LP, 10 min LP, and 20 min LP in the PFH (*n* = 80 ± 5 neurons, *n* = 85 ± 7 and *n* = 78 ± 8), LH (*n* = 4240 ± 125; 4180 ± 220; 4030 ± 185) and DMH (*n* = 90 ± 6 neurons, *n* = 87 ± 8 and *n* = 93 ± 4; Figure S1).

### 3.3 Eye Blue Light Stimulation Activates Retinal Projections to OX‐A Neurons

We hypothesized that c‐Fos activation in OX‐A neurons could be induced by the excitatory drive resulting in glutamate release from the VGluT1‐positive ipRGCs projection via the l‐RHT since these cells are specifically excited by blue light (Provencio et al. 2000, 2002; Hannibal et al. 2002; Berson et al. 2002; Warren et al. 2003). To support this hypothesis, we performed a mono‐ocular intravitreal injection of Fluo4‐Dextran (Fluo4‐Dex), a conjugated low‐affinity calcium indicator suitable to label pre‐synaptic fibers and quantify changes in pre‐synaptic Ca^2+^ flux, both in brain slices and in vivo (Kreitzer and Regehr 2001).

**FIGURE 3:**
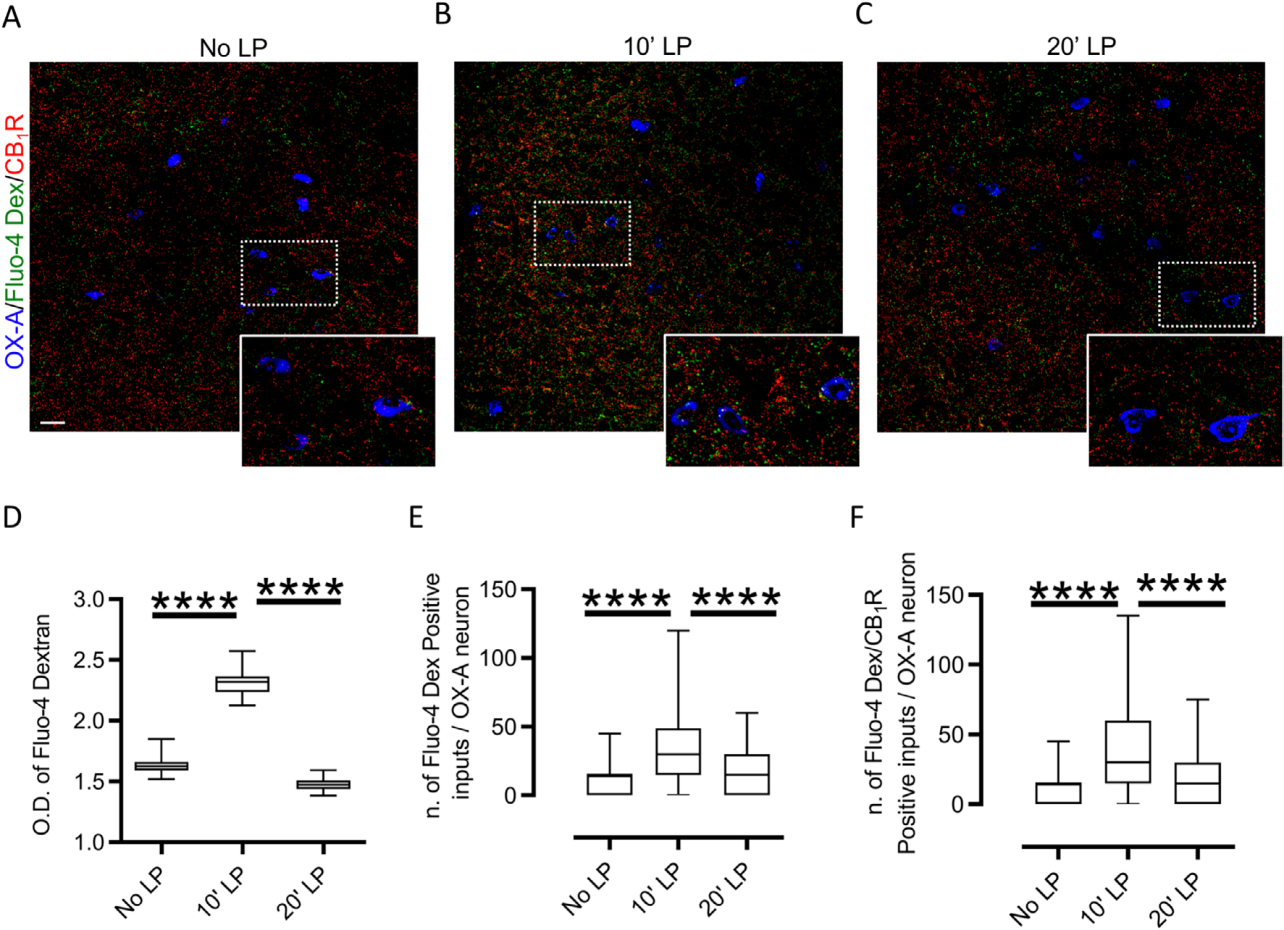
Blue light exposure activates Fluo4‐Dex/CB1R‐positive inputs to OX‐A neurons. (A–C) Confocal microscopy showing Fluo‐4‐Dx (green) and CB1R (red) colocalization in OX‐A neurons (blue) of the PFH. High magnification of the boxed areas is depicted at high magnification in each respective inset. Scale bar: 20 μm. (D) Optical density of Fluo‐4‐Dx inputs to OX‐A neurons in the PFH of mice under No LP, 10′LP or 20′LP blue light pulse exposure; *n* = 30 sections/mouse; *n* = 6 mice/group; Kruskal–Wallis Test and Dunn's post hoc test, Kruskal–Wallis statistic = 235.5, DF = 2, ****p < 0.0001; data are mean ± SEM. (E) Number of Fluo‐4‐Dx‐positive inputs to OX‐A neurons in the PFH of mice under No LP, 10′LP or 20′LP blue light pulse exposure; *n* = 30 sections/mouse; *n* = 6 mice/group; DF = 2, Kruskal–Wallis Test and Dunn's post hoc test, Kruskal–Wallis statistic = 186.2, ****p < 0.0001; data are mean ± SEM. (F) Number of Fluo4‐Dx/CB1R‐positive inputs to. OX‐A neurons in the PFH of mice under No LP, 10′LP, or 20′LP blue light pulse exposure; *n* = 30 sections/mouse; *n* = 6 mice/group. Kruskal–Wallis Test and Dunn's post hoc test, Kruskal–Wallis statistic = 199.4, DF = 2, ****p < 0.0001; data are Mean ± SEM.

Quantification of Fluo4‐Dex‐positive inputs contacting OX‐A neurons was performed in mice after 10 or 20 min of retinal stimulation with a blue light pulse. Accordingly, in addition to the 2‐AG level (Figure 1F) and c‐Fos expression (Figure 2D) pattern, we observed a change of Fluo4‐Dex fluorescence intensity in a time‐dependent response to blue light exposure (Figure 3A–D).

Specifically, the RHT fibers projecting to OX‐A neurons exhibited an increase in Fluo‐4‐Dex optical density 10 min after LP (O.D. = 2.315 ± 0.009) as compared to mice under No LP (O.D. = 1.631 ± 0.006), or after 20 min exposure to LP (O.D. = 1.477 ± 0.005) (Figure 3D). Furthermore, an increase in the number of Fluo‐4‐Dex‐positive inputs to OX‐A neurons was observed in mice after 10 min of LP (39.17 ± 1.8) compared to mice under No LP (10.91 ± 0.7), or after 20 min LP (16.11 ± 1.1) (Figure 3A–C,E).

Finally, we found that the large majority of these inputs also carried CB1R and underwent a similar time‐lapse‐dependent change of Fluo‐4‐Dex fluorescence at 10 min of LP (43.67 ± 1.8), as compared to No LP (12.62 ± 0.8) or 20 min LP (17.61 ± 1.3) (Figure 3F).

### 3.4 Eye Blue Light Stimulation Modulates the Neuronal Firing in the Lateral Hypothalamus

Previous studies based on tract‐tracing approaches have demonstrated l‐RHT is formed by ipRGCs projections to the LH (Leak and Moore 1997; Hattar et al. 2006; Delwig et al. 2016). Our data dissected the anatomical features of this branch by identifying OX‐A neurons of the PFH as targets (Figure 3E). Furthermore, by exploiting the calcium‐sensor fluorescent dye Fluo4‐Dextran, we unraveled a time‐dependent blue light‐induced calcium flux at the Fluo‐4‐Dex/CB1‐positive projections to OX‐A neurons (Figure 3F), in concomitance with c‐Fos activation of the targeted OX‐A neurons (Figure 2D) and the enhancement of 2‐AG levels (Figure 1F).

**FIGURE 4:**
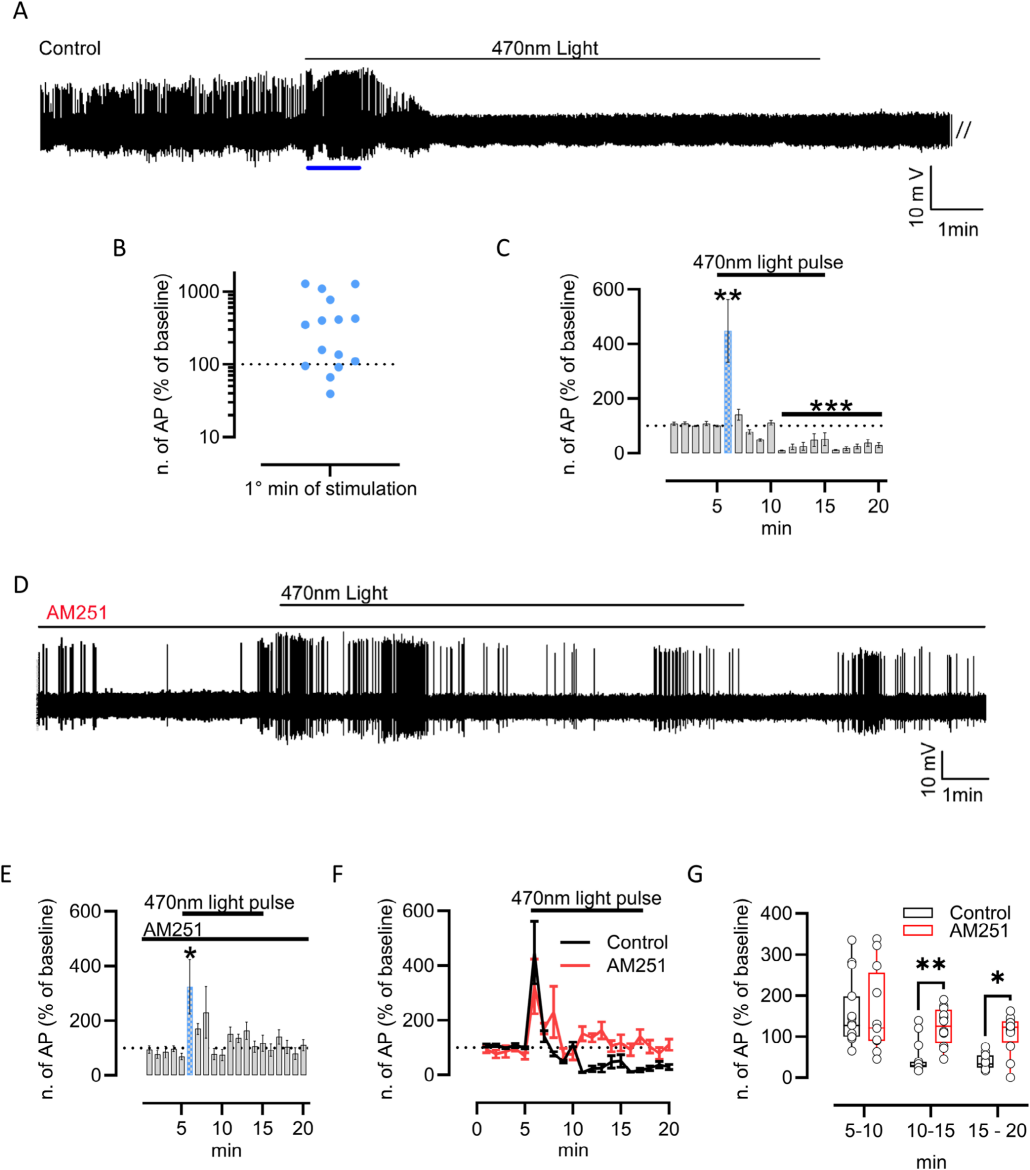
Eye Blue light exposure modulates hypothalamic neuronal activity via CB1R. (A) Representative trace and experimental design. After 5 min of baseline recording, 470 nm blue light stimulation was applied to the eyes for 10 min before recording for another 5 min in the dark condition. The blue line indicates the first minute of recording during eye stimulation. (B) Number of action potentials (AP) recorded during the first minute of stimulation expressed as a percentage of the baseline (dotted line). (C) Number of action potentials recorded during stimulation is expressed as a percentage of the baseline. *n* = 15 neurons from *n* = 10 mice, *p < 0.05 and **p < 0.01, at *t*_6min_ = two‐tailed one‐sample *T*‐Test, *t* = 3.044, df = 14, *p* = 0.0088; at *t*_11min_ = two‐tailed one‐sample *T*‐Test, *t* = 48.53, df = 14, *p* < 0.001. The blue bar underlines the percentage of the action potential in the first minute of recording during light stimulation. (D) Representative trace and experimental design. (E) Number of action potentials recorded during stimulation expressed as a percentage of the baseline; *n* = 12 neurons from 8 mice, *t*_6min_ = two‐tailed one‐sample *T*‐Test, *t* = 2.246, df = 11, *p* = 0.0462. The blue bar represents the first minute of recording during light stimulation. (F) AP frequency trend in control (black line) and AM251‐treated mice (red line). (G) Box plot comparing AP frequency over time between control (black box plot) and AM251‐treated mice (red box plot). Control, *n* = 15 neurons from 10 mice; AM251, *n* = 12 from 8 mice. 2‐way ANOVA with Sidak post hoc test. In particular, the ANOVA: Time F (DFn 2, DFd 75) = 14.54 *p* < 0.0001, Treatment F (DFn 1, DFd 75) = 13.87 *p* = 0.0004, Interaction F (DFn2, 75) = 3.092 *p* = 0.0512; Šídák's multiple comparisons test = **p < 0.01 and *p < 0.05. *t*_10–15_; *p* = 0.0033; *t*_15–20_; *p* = 0.0139.

To investigate the functional role of 2‐AG in this circuit, we conducted an in vivo single‐unit recording of unidentified neurons in the LH of mice under urethane anesthesia. Baseline firing activity was recorded for 5 min in darkness, followed by 10 min of light pulse (LP) (Figure 4A). Among the *n* = 15 neurons recorded in n=10 mice, more than 50% exhibited a six‐fold increase in firing rate in response to blue light stimulation (Figure 4B). However, all the recorded cells showed significant inhibition of their firing rate after 1 min of stimulation, a suppression that persisted even after the blue light was turned off (Figure 4C). Based on our previous observation of CB1R expression at VGluT1/CTβ‐positive RHT fibers in the PFH (Figure 1E), as well as the observed increase of 2‐AG levels following blue light stimulation (Figure 1F), we repeated the in vivo single‐unit recording after i.p. injection of AM251, a selective CB1R antagonist (4 mg/kg) (Figure 4D). Despite an initial increase in firing rate during the first minute of recording under eye blue light exposure, AM251 administration was able to prevent the subsequent suppression of firing rate. (Figure 4E, *n* = 12 cells in 8 mice). Consistent with this observation, the neuronal activity in the PFH of mice treated with AM251 was higher than in the untreated control group (Figure 4F,G).

## 4 Discussion

The ipRGCs projecting to the LH through the l‐RHT are primarily located in the upper quadrant of the retina and represent a homogeneous subset of type III ganglion cells (Canteras et al. 2011). These cells play a key role in mediating cognitive, physical, and behavioral functions influenced by blue light wavelengths, although they are not involved in the image‐forming processes (Stuber and Wise 2016). Multiple studies indicate that both natural and artificial light can affect behavior, with blue light specifically regulating arousal (Vandewalle et al. 2009; Liu et al. 2020). Consequently, blue light exposure is emerging as a potential non‐pharmacological treatment for promoting structural and functional recovery following mild traumatic brain injury (Srisurapanont et al. 2021; Bajaj et al. 2017), or psychophysiological disorders related to seasonality, such as Seasonal Affective Disorder (S.A.D) (Strong et al. 2009). However, the mechanism by which blue light regulates brain activity is not fully understood.

Previous studies have demonstrated that exposure to blue light increases c‐Fos expression in orexin/hypocretin neurons (Liu et al. 2020; Adidharma et al. 2012), one of the two main cellular subgroups in the LH regulating light‐dependent activities like sleep/wake and arousal (Stuber and Wise 2016). Consistent with these findings, our current observations indicate that short‐time lapse stimulation of ipRGCs through this selective wavelength triggers c‐Fos expression in OX‐A neurons mainly in the PFH. This selective activation was confirmed by the increase in the optical density and in the number of Fluo4‐Dex‐positive puncta in OX‐A neurons.

In this study, we examined the expression of CB1Rs among the RHT fibers projecting to OX‐A neurons in the PFH, which are affected by blue light stimulation. Our findings indicate that RHT fibers innervating OX‐A neurons in the PFH express both CB1R and VGluT1, and that blue light stimulation of these fibers induces a transient increase in 2‐AG levels, likely linked to the OX‐A neuronal activity as demonstrated by the concomitance of c‐Fos induction of expression in most of OX‐A neurons innervated by Fluo4‐Dex‐positive fibers.

In nocturnal rodents, orexin levels exhibit an increase during the dark period, coinciding with their active phase (i.e., ZT13‐24). Conversely, orexin levels decrease during the light period, reaching their lowest levels between ZT6 and ZT12 (Yoshida et al. 2001; Fujiki et al. 2001; Desarnaud et al. 2004). We conducted our experiment between ZT6 and ZT12, when orexinergic neurons are less active (Lee et al. 2005; Marston et al. 2008), to maximize the effects of our stimulation.

Notably, the reduction in 2‐AG after 20 min of blue light pulse despite the higher 2‐AG levels observed at 10 min (i.e., at 10 min LP), may reflect a shift of the neuronal activity from being depolarized to becoming hyperpolarized (20 min LP) according to change of the firing activity and of fluorescence intensity of the Fluor‐4‐Dex‐positive inputs to OX‐A neurons.

Blue light stimulation of the eye with 10 min LP triggers a transient increase in the neuronal discharge of LH neurons, followed by a long‐lasting suppression of their activity at 20 min LP. This second phase was reversed by the selective CB1R antagonist AM251, suggesting that this occurs through retrograde 2‐AG signaling at pre‐synaptic VGluT1/CB1Rs inputs, followed by inhibition of glutamate release by depolarization‐induced suppression of excitation (DSE). Interestingly, it has been shown that 2‐AG‐mediated LTD requires pre‐synaptic activity in target afferents, independently of the properties of these afferents to trigger or not a 2‐AG release (Heifets and Castillo 2009). Therefore, while the primary source of excitation in our experiment originates from the RHT fibers activated by blue light eye exposure, the subsequent increase of 2‐AG levels could induce LTD in the nearest excitatory fibers not directly activated by the blue light stimulation. This 2‐AG‐mediated paracrine effect may lead to an overall CB1R‐mediated decrease in excitatory synaptic activity, ultimately resulting in a complete cessation of electrical activity in the recorded cells.

Conversely, in the presence of AM251, we observed a transient increase in the action potential discharge, suggesting the involvement of additional homeostatic plasticity mechanisms. Despite an initial increase in firing rate during the first minute of recording following eye blue light exposure, AM251 administration was able to prevent the firing rate suppression possibly because in the healthy brain, neuronal excitability and synaptic strength are finely regulated to maintain network activity within physiological limits (Lignani et al. 2020). This regulation may explain why, despite the initial increase in firing frequency, the effect was not sustained over time but diminished by returning to baseline levels. Another factor to consider when interpreting electrophysiological results is the possibility that the observed phenomena may reflect a depolarization block. This condition involves an initial increase in action potential frequency, followed by a gradual decrease in amplitude, ultimately resulting in the cessation of spiking. This pattern is commonly linked to strong depolarization, often driven by calcium channel activation and a diminished sodium ion driving force.

Our previous findings also support this aforementioned mechanism of 2‐AG‐mediated silencing of neuronal activity, as we observed a higher number of/VGluT1/CB1R‐positive puncta per OX‐A neuron, as compared to the CB1R/VGAT‐positive ones (Cristino et al. 2013).

Our study was conducted in *Mus musculus* , a nocturnal animal. Therefore, our findings should ideally be compared to diurnal rodent models such as *Arvicanthis niloticus* , which are also used to study circadian rhythm‐related mechanisms. Several studies have highlighted differences in the activity of orexin neurons between diurnal and nocturnal species, indicating that further experiments are required to explore this aspect (Azeez et al. 2018; Martínez et al. 2002; Ikeno and Yan 2018).

To further deepen the experimental results shown in this work, we propose conducting juxtacellular recording‐labeling and immunohistochemical identification of orexin neurons to highlight potential features in their firing responses to eye blue light stimulation. On the other hand, although we did not proceed with the identification of the recorded neurons in the LH in this study, we observed a surprising homogeneity in the pattern of responses to eye stimulation.

Therefore, we considered that the anatomical and functional results obtained could reasonably allow us to discuss the effect of the eye blue light stimulation on the selective activity of the ipRGC‐derived fibers forming the l‐RHT and projecting to OX‐A neurons.

Nonetheless, the results of these experiments are consistent with the change of 2‐AG levels and Fluo‐4‐dextran optical density, suggesting that the phenomena observed under blind conditions during electrophysiological recordings could also reasonably occur in OX‐A neurons within the PFH, as indicated by c‐Fos activity.

Overall, our findings lay the groundwork for future research to clarify other aspects of the impact of blue light on the brain, including its role in regulating the sleep/wake cycle, arousal, and its effects on food intake and mood.

Future research should investigate whether and how blue light‐induced activation of the RHT influences metabolic pathways and appetite regulation. This could provide valuable insights into light‐mediated metabolic adaptations, particularly in shift workers and individuals exposed to artificial lighting at night. Indeed, growing evidence suggests that excessive or inappropriate night‐time exposure to blue light may have detrimental effects on cognitive and physiological functions. Our findings support the importance of orexinergic and endocannabinoid signaling in antidepressive non‐pharmacological therapies, although further studies need to define the interplay of the two systems at the molecular level.

## Author Contributions

**Nicola Forte:** data curation, investigation, methodology, writing – original draft, formal analysis. **Roberta Imperatore:** data curation, formal analysis, investigation, methodology, writing – original draft. **Brenda Marfella:** methodology, formal analysis. **Alessandro Nicois:** methodology, formal analysis. **Roberta Verde:** methodology, formal analysis. **Letizia Palomba:** methodology, formal analysis. **Vincenzo Di Marzo:** funding acquisition, writing – review and editing, supervision. **Luigia Cristino:** conceptualization, funding acquisition, project administration, writing – review and editing, supervision, resources.

## Disclosure

Competing interests: The authors declare that the research was conducted in the absence of any commercial or financial relationships that could be construed as a potential conflict of interest. Some of the authors were on the editorial board member of Frontiers at the time of submission. This had no impact on the peer review process, and the final decision on other papers.

## Ethics Statement

Experiments were performed following the European Union animal welfare guidelines [European Communities Council Directive of September 22, 2010 (2010/63/EU)] and the Italian Decree no. 26/2014.

## Conflicts of Interest

The authors declare no conflicts of interest.

## Supporting information


**Figure S1.** Bar graph showing the number of OX‐A neurons counted in the PFH, LH, and DMH. Data are presented as mean ± SEM.

## Data Availability

The raw data supporting the conclusion of this article will be made available by the authors, without undue reservation.
