# Supplementary material for: The Blue Light‐Responsive Lateral Pathway of the Retinohypothalamic Tract Promotes Endocannabinoid‐Driven Modulation of Orexin Neurons
Source: J Neurochem. 2025 Jun 21;169(6):e70137. doi: 10.1111/jnc.70137 (PMC12181770; doi:10.1111/jnc.70137)
Supplement: Supplementary file 1 — Figure S1. Bar graph showing the number of OX‐A neurons counted in the PFH, LH, and DMH. Data are presented as mean ± SEM. [file JNC-169-0-s001.pdf]

The blue light-responsive lateral pathway of the retinohypothalamic tract promotes endocannabinoid-driven modulation of orexin neurons

Nicola Forte[1,†], Roberta Imperatore[2,†], Brenda Marfella[1,3], Alessandro Nicois[1,4], Roberta Verde[1], Letizia Palomba[1,4], Vincenzo Di Marzo[1,5,*], Luigia Cristino[1,*]

[1]Institute of Biomolecular Chemistry, National Research Council, Via Campi Flegrei 34, 80078 Pozzuoli (NA), Italy;
[2]Department of Sciences and Technologies, University of Sannio, Via F. De Sanctis snc, 82100, Benevento, Italy;
[3]Department of Biology, University of Naples Federico II, 80126 Naples, Italy;
[4]Department of Biomolecular Sciences, University of Urbino Carlo Bo, Urbino, Italy;
[5]Canada Excellence Research Chair on the Microbiome-Endocannabinoidome Axis in Metabolic Health, Université Laval, Québec City, QC 61V0AG, Canada;
[6]Heart and Lung Research Institute of Université Laval, Québec City, QC, Canada;
[7]Institute for Nutrition and Functional Foods, Centre NUTRISS, Université Laval, Québec City, QC, Canada;

[†, *] These authors contributed equally to this work.

Correspondence: luigia.cristino@icb.cnr.it ; vincenzo.dimarzo@criucpq.ulaval.ca

Keywords: Hypocretin, chronoconnectivity, endocannabinoids, hypothalamus, retinal ganglion cells

This work is dedicated to the loving memory of Prof. Denis Richard, deceased in December 2023.

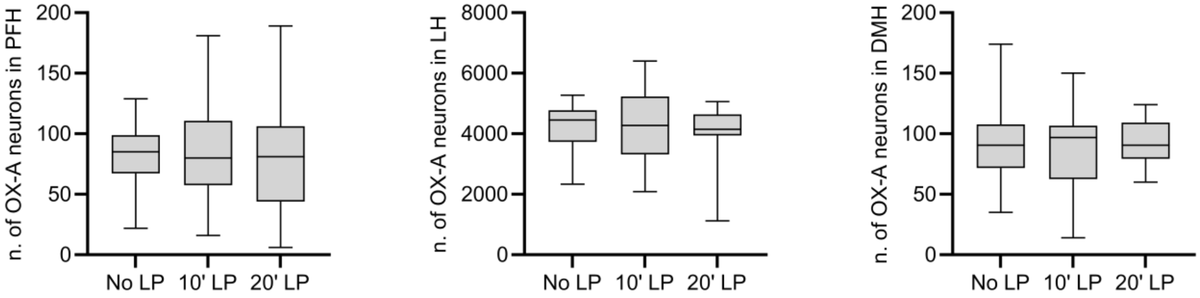

Supplementary Figure. Box plot showing the number of OX-A neurons counted in the PFH, LH, and DMH respectively.
